# Dalbergioidin Ameliorates Doxorubicin-Induced Renal Fibrosis by Suppressing the TGF-*β* Signal Pathway

**DOI:** 10.1155/2016/5147571

**Published:** 2016-12-22

**Authors:** Xianguo Ren, Yun Bo, Junting Fan, Maosheng Chen, Daliang Xu, Yang Dong, Haowei He, Xianzhi Ren, Rong Qu, Yulian Jin, Weihong Zhao, Changliang Xu

**Affiliations:** ^1^National Clinical Research Center of Kidney Diseases, Jinling Hospital, Nanjing University School of Medicine, Nanjing, China; ^2^Department of Pediatrics, Jinling Hospital, Nanjing University School of Medicine, Nanjing, China; ^3^Department of Geriatrics, The First Affiliated Hospital of Nanjing Medical University, Nanjing, China; ^4^School of Pharmacy, Nanjing Medical University, Nanjing, China; ^5^Department of Nephrology, Zhejiang Provincial People's Hospital, Hangzhou, China; ^6^Department of Nephrology, Anhui Provincial Children's Hospital, Hefei, China; ^7^Nanjing University of Traditional Chinese Medicine, Nanjing, China; ^8^Department of Urology, Jinling Hospital, Nanjing University School of Medicine, Nanjing, China

## Abstract

We investigated the effect of Dalbergioidin (DAL), a well-known natural product extracted from* Uraria crinita*, on doxorubicin- (DXR-) induced renal fibrosis in mice. The mice were pretreated for 7 days with DAL followed by a single injection of DXR (10 mg/kg) via the tail vein. Renal function was analyzed 5 weeks after DXR treatment. DXR caused nephrotoxicity. The symptoms of nephrotic syndrome were greatly improved after DAL treatment. The indices of renal fibrosis, the phosphorylation of Smad3, and the expression of alpha-smooth muscle actin (*α*-SMA), fibronectin, collagen III (Col III), E-cadherin, TGF-*β*, and Smad7 in response to DXR were all similarly modified by DAL. The present findings suggest that DAL improved the markers for kidney damage investigated in this model of DXR-induced experimental nephrotoxicity.

## 1. Introduction

Doxorubicin (DXR) is an anthracycline glycoside antibiotic that has broad-spectrum antitumor activity against a variety of human solid tumors—such as ovarian, breast, and lung cancers—as well as several other cancers and hematologic malignancies [1–3]. However, DXR does not discriminate between cancer and normal cells and eradicates not only fast-growing cancer cells but also other rapidly growing cells in the body; therefore, its use in chemotherapy has been restricted. DXR has a variety of toxicities, including cardiac, hepatic, renal, and hematologic toxicity [4–8]. Although the mechanism underlying the severe cytotoxicity from DXR is not fully understood, reactive oxygen species (ROS) are assumed to be a key factor. It is very important to understand the events controlling this oxidative injury. DXR treatment leads to the overproduction of hydroxyl radicals, hydrogen peroxide, and superoxide anions, which cause membrane lipid peroxidation [9]. Therefore, increasing data suggest that simultaneous treatment with DXR and an antioxidant may alleviate the toxicity of DXR.

*Uraria crinite*, which has some great health benefits, is widely distributed throughout India, Thailand, Indonesia, and China. It has long been used as a herbal medicine, having bioactive properties, such as antioxidant activity, antiulcer effects, and osteogenic activity. Its roots, because of their anti-inflammatory activity, have also been used to treat chills, edema, and stomachache [10, 11]. The aim of the present study was to investigate the effect of Dalbergioidin (DAL), a well-known anthocyanin from* Uraria crinita*, on DXR-induced renal fibrosis in mice. The study was performed to determine whether treatment with DAL could counteract renal fibrosis induced by DRX in vivo. We also investigated DAL's mechanism of action.

## 2. Methods

### 2.1. Reagents

DAL was purchased from BioBioPha Co., Ltd. SMAD3, p-SMAD3, SMAD7, *α*-SMA, fibronectin, Col I, E-cadherin, and TGF-*β* were purchased from Santa Cruz Biotechnology, Inc. Bovine serum albumin (BSA), DXR, sodium hydroxide, ferric nitrate, trichloroacetic acid (TCA), and perchloric acid (PCA) were obtained from Sigma-Aldrich.

### 2.2. Animals

The mice were housed and used as previously described [12].

### 2.3. Experimental Procedure

The mice were randomly divided into 3 groups of 8 mice each. Group I served as the control group for 42 days. Group II served as the model group and received a single IV injection of DXR (10 mg/kg) on day 7. Group III served as the treatment group and was pretreated with DAL (30 mg/kg IP) for 42 days; on day 7, a single IV injection of DXR (10 mg/kg) was administered. On day 42, the mice were sacrificed by cervical dislocation and, after perfusion to evaluate the various biochemical parameters, kidney and blood samples were taken.

### 2.4. Measurement of Urine and Plasma

Urine and blood samples were collected as previously described [13]. Urine albumin, plasma triglyceride levels, plasma urea levels, and serum creatinine levels were determined using commercial kits, an enzyme-linked immunosorbent assay kit (Exocell), a Urea Nitrogen Direct Kit (Stanbio Laboratory), a LabAssay Triglyceride ELISA Kit (Wako), and a Creatinine Liquicolor Kit (Stanbio Laboratory).

### 2.5. Masson-Trichrome Staining

Masson-trichrome staining was done as previously described [14].

### 2.6. Determination of GSH In Vivo

The effect of DAL treatment on Glutathione (GSH) levels was evaluated using a commercial kit (Cayman Chemical Co.) following the manufacturer's protocol.

### 2.7. Determination of MDA Levels In Vivo

The lipid peroxidation of the kidney tissue was studied by measuring the malondialdehyde (MDA) levels in a colorimetric method involving thiobarbituric acid (TBA) adduct formation. MDA was measured by a commercial TBARS Assay Kit (Cayman Chemical Co.) following the manufacturer's protocol.

### 2.8. Reverse Transcription Polymerase Chain Reaction (RT–PCR)

Total RNA was isolated from the cells using a commercial TRIzol reagent kit (Invitrogene); the RNA concentrations were measured spectrophotometrically. The first cDNA synthesis was performed following the manufacturer's instructions (Takara, JPN). The specific primers for fibronectin, *α*-SMA, E-cadherin, Col III, SMAD7, TGF-*β*, and GAPDH (loading control) were as follows:

fibronectin: sense 5′-CGAGGTGACAGAGACCACAA-3′, antisense 5′-CTGGAGTCAAGCCAGACACA-3′; *α*-SMA: sense 5′-TGTGCTGGACTCTGGAGATG-3′, antisense 5′-ATGTCACGGACAATCTCACG-3′; E-cadherin: sense 5′-AATGGCGGCAATGCAATCCCAAGA-3′, antisense 5′-TGCCACAGACCGATTGTGGAGATA-3′; Col III: sense 5′-AGGCAACAGTGGTTCTCCTG-3′, antisense 5′-GACCTCGTGCTCCAGTTAGC-3′; smad7: sense 5′-AGGTGTTCCCCGGTTTCTCCA-3′; antisense: 5′-TTCACAAAGCTGATCTGCACGGT-3′; TGF-*β*: sense 5′-GCAACATGTGGAACTCTACCAGAA-3′, antisense 5′-GACGTCAAAAGACAGCCACTCA-3′; GAPDH: sense 5′-AACTTTGGCATTGTGGAAGG-3′, antisense 5′-ACACATTGGGGGTAGGAACA-3′. The protocol was as follows: 50°C for 2 minutes, 95°C for 10 minutes, 40 cycles of 95°C for 15 seconds, and 60°C for 30 seconds.

### 2.9. Western Blot Analyses

Using the western blotting method as previously described [12], the tissues were homogenized and the supernatant was then decanted. First antibodies were added and incubated with membranes at 4°C overnight. HRP-conjugated secondary antibodies were diluted and incubated with the membranes at 20°C. The blots were then incubated with a chemiluminescent substrate (Millipore) and exposed to Kodak Film.

### 2.10. ELISA Assay

TGF-*β* was measured using a TGF-*β* ELISA Quantitation Kit following the manufacturer's protocol (R & D, Inc.).

### 2.11. Statistical Analysis

Differences between the groups were analyzed by Student's *t*-test. All the data points are presented as the treatment group's mean  ±  standard deviation (SD) of the mean. *p* values less than 0.05 were considered significant.

## 3. Results

### 3.1. Effect of DAL on Renal Dysfunction

**Figure 1:**
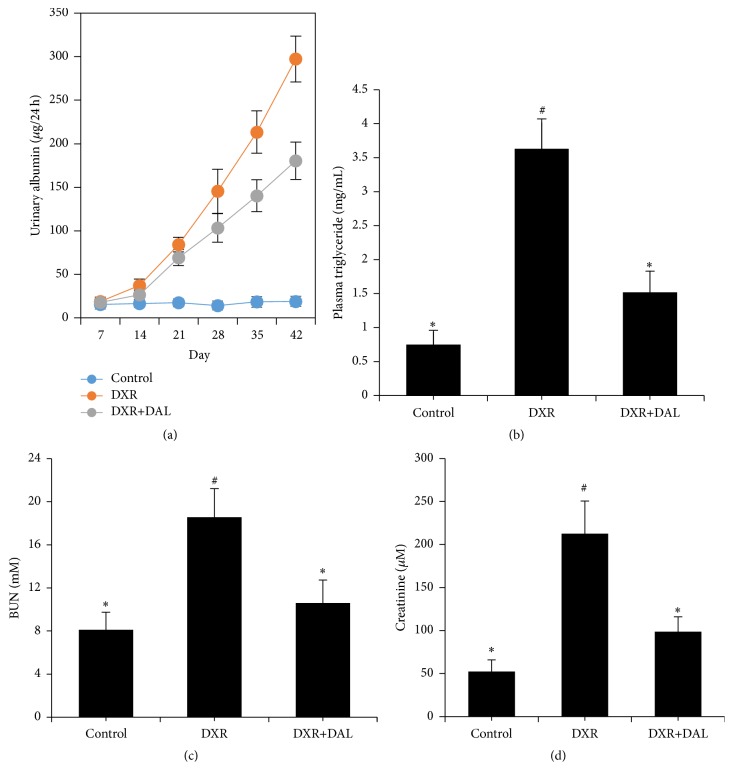
Kidney injury at 5 weeks after DXR injection in different groups of mice as indicated. (a) Effect of DAL on albuminuria against DXR-induced nephrotoxicity; (b) effect of DAL on hyperlipidemia against DXR-induced nephrotoxicity; (c) effect of DAL on blood urea nitrogen (BUN) against DXR-induced nephrotoxicity; (d) effect of DAL on serum creatinine against DXR-induced nephrotoxicity. The control and DAL treatment groups are compared with the DXR group. Values are statistically significant at ^*∗*^ *p* < 0.05; the DXR group is compared with the control group. Values are statistically significant at ^#^ *p* < 0.05.

As shown in Figure 1(a), the 24-hour urinary protein excretion of the mice progressively increased after the injection of DXR. On day 21, the urinary protein of the DXR-treated mice was significantly higher than that of the control mice. Beginning on day 28, the urinary protein of DXR-treated mice rapidly increased. Treatment with DAL significantly decreased urinary protein at the weeks 5 and 6. The DXR-treated mice developed severe hyperlipidemia (plasma triglyceride 3.63 ± 0.44 mg/mL), which was less severe in the treatment group (plasma triglyceride 1.52 ± 0.31 mg/mL) (Figure 1(b)). The treatment of mice with DXR caused a significant increase in BUN and plasma creatinine levels by 2.3- and 4.1-fold, respectively, compared with the control group (Figures 1(c) and 1(d)). Pretreatment with DAL for 7 days resulted in the restoration of BUN and plasma creatinine to near control levels (*p* < 0.01). Therefore, DAL attenuates nephrotoxicity in a mouse model of DXR.

### 3.2. Effect of DAL on Renal Fibrosis

**Figure 2:**
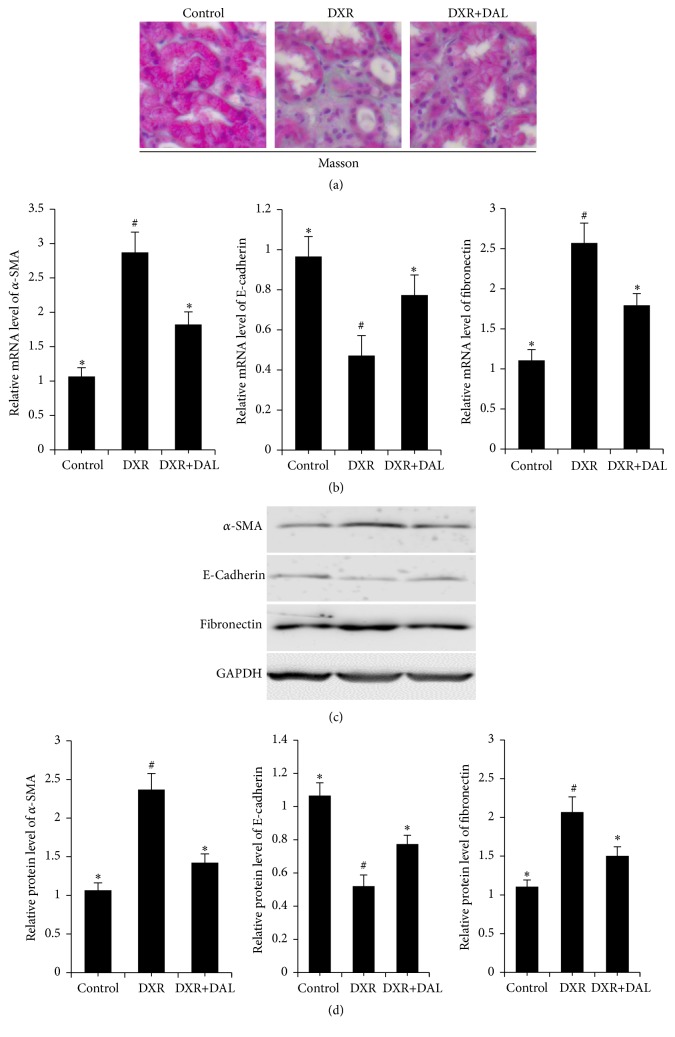
Renal fibrosis at 5 weeks after DXR injection in different groups of mice as indicated. (a) Kidney sections were subjected to Masson-trichrome staining; (b) kidney sections expressed the mRNA of *α*-SMA, and E-cadherin, fibronectin; (c) kidney sections expressed the protein of *α*-SMA, E-cadherin, and fibronectin; (d) the relative protein expression of fibronectin, *α*-SMA, and E-cadherin as seen in the kidney sections. The control and DAL treatment groups are compared with the DXR group. Values are statistically significant at ^*∗*^ *p* < 0.05; the DXR group is compared with the control group. Values are statistically significant at ^#^ *p* < 0.05.

Like many other organ systems, the kidney stiffens after injury, a process increasingly recognized as an important driver of renal fibrosis [15]. To correlate the reduction of kidney injury with the effect of the drug treatments, renal fibrosis was assessed by Masson staining (Figure 2(a)). The renal fibrosis marker of alpha-smooth muscle actin (*α*-SMA), fibronectin, and the epithelial cell marker of E-cadherin were assessed by western blotting [16, 17]. Consistent with the albuminuria data, the results from the DXR mice showed marked renal fibrosis, as evidenced by the increased expression of fibroblasts markers (Figures 2(b)–2(d)). The treatment of mice with DXR caused a significant increase in the renal protein expression of well-known fibroblasts markers and increased the expression of E-cadherin in renal tissue (Figures 2(b)–2(d)). DAL ameliorates renal fibrosis in a mouse model of DXR.

### 3.3. Effect of DAL on Kidney Redox Potential

**Figure 3:**
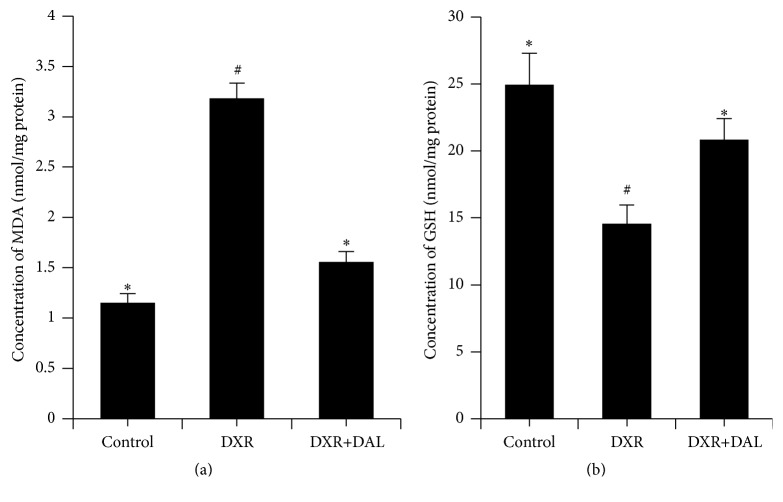
Redox microenvironment in kidney tissue at 5 weeks after DXR injection in different groups of mice as indicated. (a) Effect of DAL on kidney MDA levels; (b) Effect of DAL on kidney GSH levels. The control and DAL treatment groups are compared with the DXR group. Values are statistically significant at ^*∗*^ *p* < 0.05; the DXR group is compared with the control group. Values are statistically significant at ^#^ *p* < 0.05.

The elevated production of reactive oxygen species (ROS) is a primary mechanism of DXR-induced cytotoxicity [18, 19]. MDA and GSH serve to assess the level of ROS. In this study, there was a significant increase of MDA in the kidneys of the DXR group compared with the control group (*p* < 0.01). Compared with the DXR group, the mice with DXR-induced nephrotoxicity that were treated with DAL showed a significant reduction in MDA levels (Figure 3(a)). We also measured the GSH concentration as an indicator of cellular redox status in the kidney tissue to investigate the antioxidant action of DAL. After the DXR treatment, the levels of GSH were significantly depleted, as shown in Figure 3(b) (*p* < 0.01). Compared with the DXR group, the group that received DAL showed a significantly reversed GSH depletion. This shows that DAL maintains the redox balance of kidney tissue.

### 3.4. DAL Effects on the TGF-*β* Signaling Pathway

**Figure 4:**
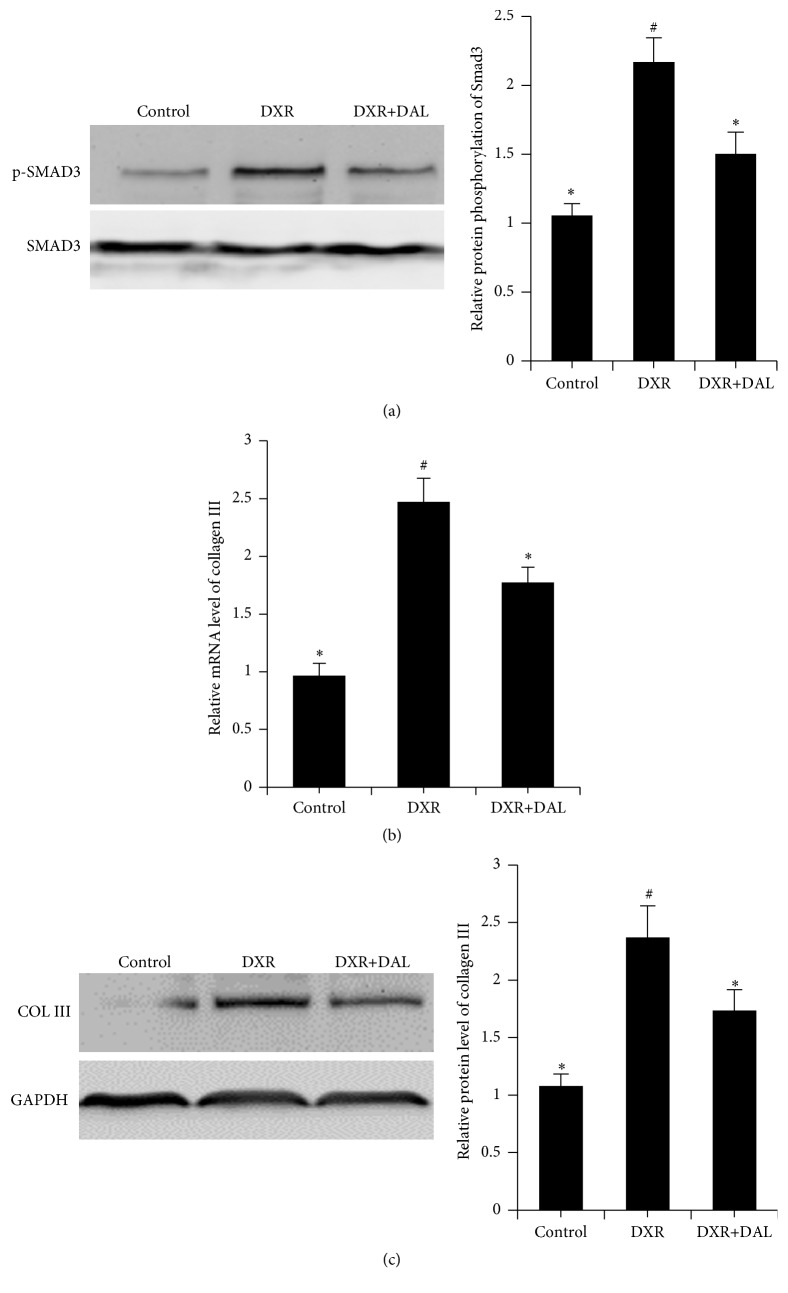
The TGF-*β* signaling pathway in kidney tissue 5 weeks after DXR injection in different groups of mice as indicated. (a) DAL inhibits the phosphorylation of Smad3; (b) DAL inhibits the gene expression of collagen III, which is the target gene of Smad3; (c) DAL inhibits the protein expression of collagen III, which is the target protein expression of Smad3. The control and DAL treatment groups are compared with the DXR group. Values are statistically significant at ^*∗*^ *p* < 0.05; the DXR group is compared with the control group. Values are statistically significant at ^#^ *p* < 0.05.

**Figure 5:**
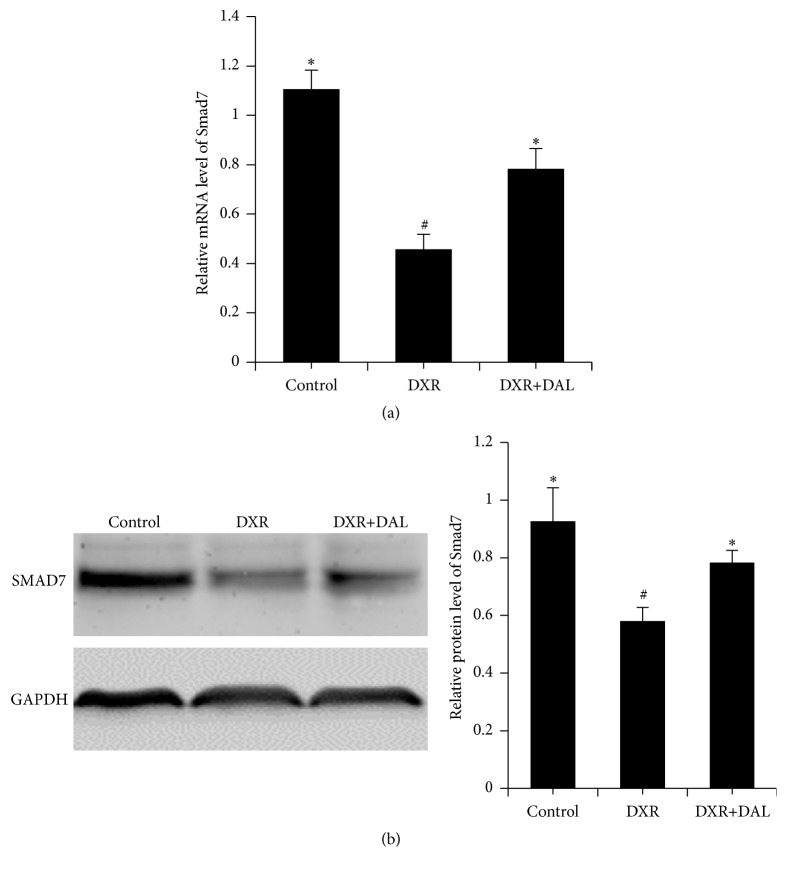
The expression of Smad7, an inhibitor of the TGF-*β* signaling pathway, in kidney tissue 5 weeks after DXR injection in different groups of mice as indicated. (a) DAL increases the gene expression of Smad7; (b) DAL increases the protein expression of Smad7. The control and DAL treatment groups are compared with the DXR group. Values are statistically significant at ^*∗*^ *p* < 0.05; the DXR group is compared with the control group. Values are statistically significant at ^#^ *p* < 0.05.

TGF-*β* is a key mediator in the pathogenesis of renal fibrosis and induces renal scarring largely by activating its downstream Smad signaling pathway [20]. Although the TGF-*β* signaling pathway is mediated by Smad2 and Smad3, Smad2 protects against TGF-beta/Smad3-mediated renal fibrosis [21]. Thus, phosphorylated Smad3 is the effector of TGF-*β*-mediated renal fibrosis. The levels of phosphorylated Smad3 in the kidney were increased by DXR (Figure 4(a)). As shown in Figure 4(a), the DAL-treated groups exhibited a significant decrease in the phosphorylation level of SMAD3 compared with the DXR groups (*p* < 0.01). Because collagen III is a target gene of TGF-beta/SMAD3, the mRNA and protein expression of collagen III in the kidney was evaluated by q-PCR and western blotting. As shown in Figures 4(b) and 4(c), collagen III mRNA and protein levels in the DAL-treated groups showed a significant decrease compared with those in the DXR groups. Smad7 acts as an antagonist of the TGF-*β* signaling pathway by preventing R-Smads from interacting with their receptors or by competing with Co-Smads for the generation of R-Smad/Co-Smad complexes [22, 23]. Smad7 mRNA and protein levels were reduced after DXR treatment, but this effect was reversed by DAL treatment (*p* < 0.01; Figures 5(a) and 5(b)). DAL suppresses the TGF-*β* signaling pathway in kidney tissue.

### 3.5. Effect of DAL on TGF-*β* Protein Expression

**Figure 6:**
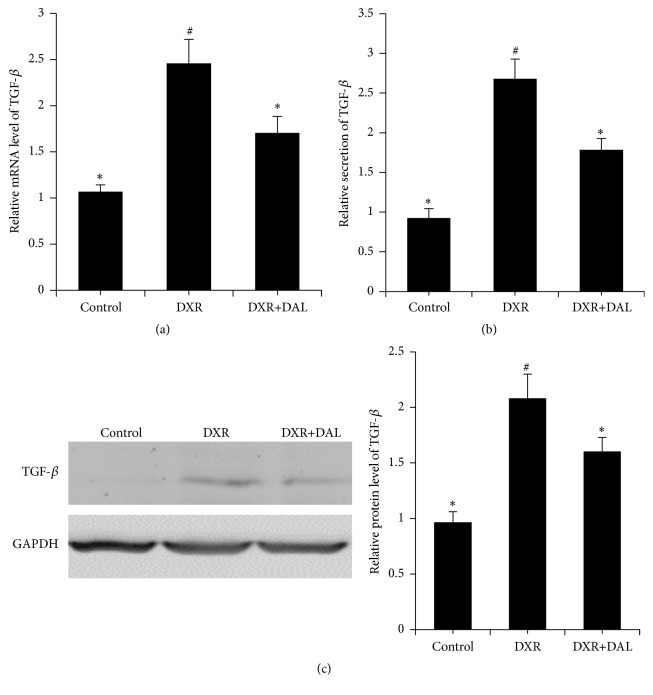
The expression of TGF-*β* in kidney tissue 5 weeks after DXR injection in different groups of mice as indicated. (a) DAL decreases the gene expression of TGF-*β*; (b) DAL decreases the protein expression of TGF-*β* by ELISA; (c) DAL decreases the protein expression of TGF-*β* as determined by western blotting. The control and DAL treatment groups are compared with the DXR group. Values are statistically significant at ^*∗*^ *p* < 0.05; the DXR group is compared with the control group. Values are statistically significant at ^#^ *p* < 0.05.

TGF-*β* is a protein that controls proliferation, cellular differentiation, and other functions in most cells. TGF-*β* is important for the induction of fibrosis and the EMT often associated with the chronic phases of inflammatory diseases [24]. As shown in Figure 6, the mRNA and protein levels of TGF-*β* in the DAL-treated groups showed a significant decrease compared with those in the DXR-treated groups.

## 4. Discussion

In the current study, DAL ameliorated the severe nephritic syndrome induced by DXR in mice. Urine albumin and plasma urea and creatinine are the most sensitive markers of nephrotoxicity implicated in the diagnosis of renal injury [25, 26]. DXR treatment significantly increased serum creatinine, BUN, and hyperlipidemia. In contrast, treatment with DAL resulted in a significant decrease of these parameters in the DXR-treated animals. These results indicate that DAL may offer a considerable nephroprotective effect against DXR toxicity.

Renal fibrosis is a well-known cause of kidney failure in DXR-induced nephropathy [27]. Several cellular pathways, including fibroblast activation and tubular epithelial-mesenchymal transition, have been identified as the major causes of renal fibrosis [28]. In this study, the administration of DAL significantly improved renal fibrosis. One of the major mechanisms in the protection of DAL in this model involves the inhibition of fibroblast activation. After 35 days of DXR injection, fibronectin and *α*-SMA mRNA and protein levels were markedly upregulated and DAL significantly inhibited their levels of expression. We also examined the mRNA and protein levels of the epithelial marker E-cadherin. DAL treatment reversed the reduction of E-cadherin. Consistent with these results histologically, DAL treatment ameliorated DXR-induced renal fibrosis.

TGF-*β*, which is upregulated in some studies, plays a pivotal role in the progression of the tubular epithelial-mesenchymal transition in renal fibrosis; therapeutic intervention targeting TGF-*β* has been successful and well tolerated in animal models [9, 21, 23]. It has recently been postulated that ROS mediate fibrosis via a TGF-*β*-dependent pathway [29, 30]. Moreover, ROS have emerged in the pathogenesis of DXR-induced nephropathy [31, 32]. It has been suggested that a DXR semiquinone plays a major role in DXR nephrotoxicity. Although semiquinones have a short life, they initiate a stream of reactions, producing ROS after interacting with molecular oxygen [5, 33]. It has been shown that DXR increases the production of free radicals such as superoxide, hydroxyl radicals, and hydrogen peroxide, which have a great ability to react rapidly with lipids and cause lipid hydroperoxide (LPO) [9]. LPO is known to be one of the toxic manifestations of DXR ingestion; its presence is determined by measuring MDA levels. Excessive LPO has been reported in the kidneys of DXR-treated mice [34]. In the current study, the DXR-treated mice showed increased levels of MDA compared with the control mice. GSH is the most important thiol-containing antioxidant, and it plays a pivotal role in preventing oxidative damage [35, 36]. GSH has also been used as a biomarker of oxidative stress in biological systems [37]. The depletion of GSH has been observed in DXR mice [34]. In our studies, DAL decreased the concentrations of MDA and increased the level of GSH. The recovering redox balance in the tissue microenvironment is the most likely mechanism by which DAL exerts nephroprotective effect and inhibits a tubular epithelial-mesenchymal transition.

Smad3 is a critical downstream mediator responsible for the biological effects of TGF-*β*, and their related family members regulate the transcription of several hundred genes. In the context of renal fibrosis, Smad3 are strongly activated in both experimental and human kidney diseases [20]. Phosphorylated Smad3 is increased in the DXR group. This observation indicates that TGF-*β*/Smad signaling pathways are activated in DXR-induced nephropathy. However, this phenomenon is reversed by DAL. Collagen I, which is a fibrogenic gene, is the downstream target of the TGF-*β*/Smad3 signaling pathway. DAL reversed the increase of collagen I. Furthermore, Smad7, which is an inhibitor of the TGF-*β*/Smad signaling pathways, was upregulated by DAL treatment. DAL also increases the mRNA and protein expression of TGF-*β* in DXR-induced nephropathy.

In conclusion, our results demonstrate that DAL has a potent nephroprotective effect in the DXR mice model. The nephroprotective effect of DAL may be mediated by suppressing the TGF-*β*-induced renal tubular epithelial-to-mesenchymal transition. This is an early-stage study of the nephroprotective effects of DAL; the detailed mechanisms of action need further clarification.
